# Error Aggregation in the Reengineering Process from 3D Scanning to Printing

**DOI:** 10.1155/2017/1218541

**Published:** 2017-11-07

**Authors:** Jennifer G. Michaeli, Matthew C. DeGroff, Roman C. Roxas

**Affiliations:** Old Dominion University, Norfolk, VA 23529, USA

## Abstract

This work aims to study the aggregation of dimensional errors in the reengineering processes using 3D scanning and printing without initial design drawings. A 57-tooth spur gear is used as an example to facilitate the discussion. Two approaches are investigated. The first one builds the gear model based upon measurement taken from a caliper, and the second approach uses a 3D scanner to collect geometry data. Dimensional errors in each stage of these two approaches are investigated. Particular attention is paid to the geometry data flow in the reengineering process from data acquisition and editing to model construction. Recommendations are made in regard to error estimation and alleviation.

## 1. Introduction

The reverse engineering process incorporating 3D scanning and 3D printing is used in a variety of fields and disciplines including engineering, medicine, oceanography, biology, and historic preservation. Medical and biology professionals, for example, used 3D scanning and printing to create dental implants, a human skull, hand orthoses, models of organs, marine organisms, coral reef, and even scale models of the embryonic stages of certain animals [1–5]. In engineering, the process has been used to create objects including aircraft parts and replacement parts for buildings [6–8]. Many of these applications are required to meet performance standards and achieve a high degree of accuracy, especially if the parts to be reverse engineered must fit with other components. Therefore, it is necessary to quantify the accuracy and reliability of the process for future progress in this area.

The importance of the reengineering process can be seen in the applications that have been researched [9–11]. These applications in literature can be summarized into two basic categories. They are creating components to be compatible with scanned objects or replicating parts exactly.

In creating compatible parts for scanned components some studies focused on detailed scans of human physiology to create biomechanical structures. Baronio et al. created a hand orthosis by scanning a human arm and hand with a scan-in-a-box light scanner and printing it on a Stratasys FDM printer [5]. The challenge presented in this was scanning and aligning a slightly moving and fluctuating object. In Joo et al.'s study, clinicians used a combination of scanning and printing to create interim dental implants for a patient [1]. Neither of these studies attempted to quantify the errors associated with the scanning or printing processes.

In the area of duplicating exact parts, Eslami et al. demonstrated the process by scanning and modeling an aircraft wing component [6]. They used a faro arm to laser scan the part. They then modeled the part using a combination of the Geomagic software suite and SolidWorks CAD software. The finished CAD model was used for FEA analysis, 3D printing of a prototype, and creating data for traditional manufacturing [6]. The error in this process, however, was not discussed. In another study, researchers demonstrated the effectiveness of the process for preserving cultural artifacts [6]. They used a Range 7 triangulation laser scanner in combination with a CraftBot FDM printer and an EnvisionTEC SLA printer. They began by determining the operating precision of the scanner to compare it to the value listed in the manual. The researchers then analyzed the accuracy of the two printers by laser scanning the artifacts from each printer and measuring them within the scanner software. From this they were able to determine the dimensional error in each printed part by comparing their measurements with that of the geometry model produced by the scanner software for printing [10]. While Balletti et al. discussed the precision of the scanner, they did not determine the error in the scans from the original objects. Error in the 3D scanning process was the focus of Yao's research [12]. In this study, he 3D printed parts from an original CAD file. He then measured the printed parts with a caliper and with a 3D scanner. The errors in measurements from both were compared to the original CAD data. In his study, he found that the errors in the scan measurements were smaller than those from caliper measurements [12].

In the reverse engineering process, errors are introduced in both the scanning and printing stages. Scanning acquires geometric data of the reengineered parts through haptic technology or optical and laser devices. The data acquisition phase generates error as well as the follow-up data processing and modeling phases. These errors can be classified as hardware limitations, numerical errors, and unreliable human judgement.

In the data acquisition phase, there may be holes or portions of missing data due to human error in not capturing all portions of the object or due to the properties of the object being scanned [8]. This error results because the operating principle is based on the scanner. The scanner must have a line of sight and be able to see the object. In the first case the error is alleviated using more scanning angles to capture the entire part. The second case may be a result of the complexities of the part geometry or the color or material of the object. Often, the color or material may absorb or reflect most of the light, which results in not enough light being received by the scanner. This can be fixed in many cases by adjusting the exposure and resolution of the camera and spraying the part with a neutral shade of paint or developer. Despite these efforts there may still be missing data in certain regions of the part.

The data processing phase may generate errors in the point editing and meshing steps. In the point cloud editing stage, individual scans must be merged, and overlap in data must be eliminated. Any misalignment or overlap can cause inaccuracies in the mesh such as unevenness [13]. The degree to which errors of this nature occur depends partly on operator skill or judgement, because the process must be performed manually. In the meshing stage, the data is represented by a surface of triangles. This necessity introduces error, because the collection of polygons can only approximate the shapes of the original object [13].

The last step in the scanning process is the modeling phase, which contains its own errors. Either the model can be constructed by creating a NURBS surface that creates a best fit over the mesh and creates smooth surfaces in place of polygonal faces [5, 13] or the model can be constructed parametrically. The parametric approach uses the mesh as a guide for the model sketches. These sketches are then used to create the model using basic CAD commands. In the case of the NURBS based model, the error is numerical. The scanning software, in many cases, calculates the best NURBS surface based on the desired tolerance or method of fit [5, 13]. Parametric modeling errors can be introduced by the operator, because the decisions made when constructing surfaces or solids based on the mesh are greatly dependent on human judgement. The software used in this study, Geomagic Design X (3D Systems, Inc., SC), shows the operator the deviation between his model and the scan data while the operator is in the process of constructing it. This allows the operator to refine his model to increase the tolerance. However, this does not fully eliminate the human error.

Finally, a physical part is created by 3D printing the model. In this process, the model is divided into layers and built on the printer layer by layer in an additive process [14–18]. Sources of error stemming from this vary depending on the printing process and the material. Printing processes include various metal printing, such as powder bed methods, and plastic printing. Two of the most popular methods are laser sintering methods, such as direct metal laser sintering (DMLS), and plastic extrusion methods, such as fused deposition modeling (FDM). Some of the printing parameters that can affect the dimensional accuracy include temperature, cooling rates after printing, build speed, and material. Many printing methods require an energy input that introduces heat into the material. In plastic extrusion based processes the heat is usually supplied by a print-head that the material is forced through [16, 18]. Metal based processes, such as DMLS, in contrast use a laser to supply heat to a bed of metal powder. The temperature must be high enough to ensure that each layer bonds well to the next; however, if the temperature is too high the melt flow can cause inaccuracies [18]. Cooling of the material after printing can also cause shrinkage or warping of the parts [16–18]. Some researchers have decreased error as a result of this by inventing adaptive scan times that increase or decrease depending on part length [14, 15] and finite element methods that allow the printer to be calibrated for shrinkage in different build directions [17]. Printing speed can also affect accuracy. In metal sintering processes, increasing scan speed can lower the percent shrinkage [17]. In extrusion based processes if the material flows too quickly, the printed part will have lower precision [18]. Additionally, the speed of the print-head must match the extrusion rate of the material or the printed part may have too much or too little material [18]. The printed materials can also affect part quality [16]. For example, in plastic 3D printers, different plastics such as ABS or PLA have different thermal coefficients. Since ABS has a high thermal coefficient, each layer may cool too much before the next layer is applied [18]. This can weaken the bonds between each layer and cause weaknesses between layers. Therefore, it is important to control the environmental temperature when printing.

The motivation for this study is to quantify the error in both scanning and printing and determine the reliability of the combined process. Reverse engineering of a 57-tooth spur gear without its design drawing is presented as an example. The gear is scanned using a Geomagic Capture and then printed using a Stratasys FDM printer. These printed gears are then measured using a caliper. The approach is then compared to the printed models based on caliper measurements of the spur gear. An efficient way to improve the results by using mean scan measurements is also suggested and validated in this study. In the printing process, the parameters of the machine used are presented in the paper. These are assumed to be the optimum printing parameters, and they are not explored as variables in the experiment.

## 2. Materials and Methods

The task we are concerned with in this study is to reverse engineer a machine component without its design drawing. One may use caliper measurements or a scanner to produce the required geometry model and then submit it for 3D printing. The error propagation or aggregation throughout this reengineering process is the focus of this study.

**Figure 1:**
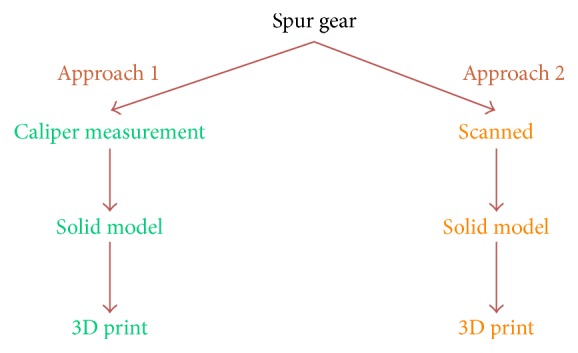
Approach 1 based on caliper measurements shown on the left branch; Approach 2 based on scanning shown on the right branch.

The error propagation in the reengineering process considered here includes two approaches each contributing its own errors. The flow chart in Figure 1 shows that with each step in the printing process error is introduced. In Approach 1 of the flowchart, the original gear is measured using a caliper, and a solid model is then drawn in CAD based on these measurements. The part is then printed from the solid model. This process includes the errors from the caliper measurements and the 3D printing. In Approach 2, the original gear is scanned, and a solid model is created in the scanner software or drawn in solid modeling software. The error in this process includes original error from the scanning step, and additional errors in the solid modeling and printing steps.

**Figure 2:**
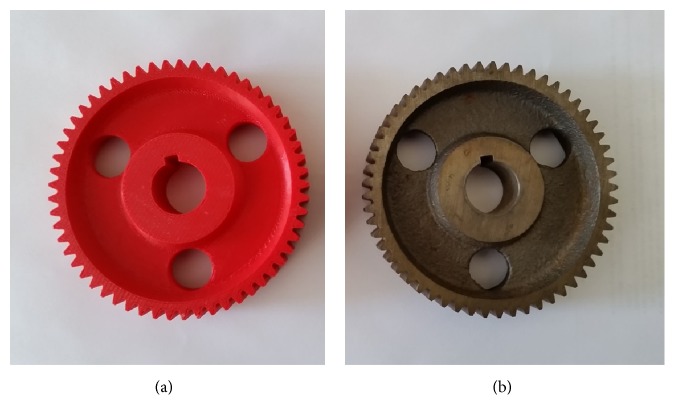
Scanned and printed gear based on average scanned tooth full depth (a); 57-tooth spur gear (b).

In this research, the 57-tooth spur gear, illustrated on Figure 2(b), was chosen because the intricacy of the tooth profile and arrangement provided a challenge for scanning and the number of teeth on each gear was sufficient to calculate an average and standard deviation for each part with confidence. The key dimensional parameter of concern in this study was the full depth of each gear tooth.

### 2.1. Scanner

**Figure 3:**
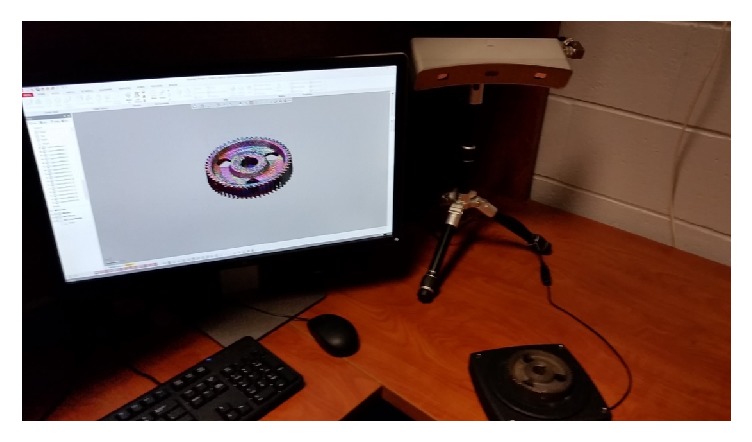
Scanner and gear experimental setup.

**Figure 4:**
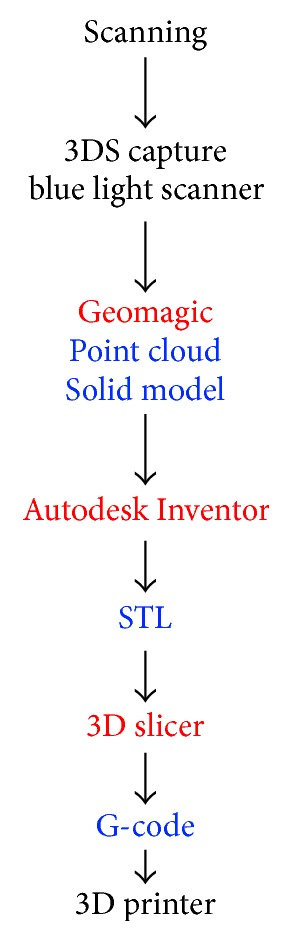
Outline of scanning and printing steps.

The scanner used in this study was a 3D Systems Capture blue light scanner (3D Systems, Inc., SC) with a turntable on which the gear could be scanned, seen in Figure 3. The scanner has an accuracy of ±0.0024 in. and a capture rate of 985,000 points/scan. In this research, the capture was used in conjunction with the software Geomagic Design X (3D Systems, Inc., SC) and Inventor (Autodesk, Inc., CA) to generate the required solid model. This scanner captured most of the features of the gear including the size of many of the teeth. The scanning workflow was based on three steps. These were data acquisition, point cloud editing, and mesh generation. During these steps, approximately 10 to 15 minutes was spent on data acquisition. This included accumulated time spent scanning from 22 angles. After data acquisition, 2 to 3 hours was spent on data processing and modeling. The modeling time varied depending on how the gear was reconstructed. In one process the gear teeth were modeled individually. In the other process, the teeth were measured after meshing, and the gear teeth were modeled off the mean dimension. The second method was less tedious and reduced the time by approximately 30 minutes to 1 hour. The operational time can change based on the level of skill or familiarity with the software. However, much time and training are required to learn how to properly and efficiently operate the scanner and software. The entire process from scanning to printing is outlined in Figure 4.

During the scanning stage, 22 scans of the gear were taken. 18 of these scans were performed with the gear bore axis perpendicular to the table and were automatically aligned through sphere registration. The purpose of these scans was to capture the gear teeth. The other 4 scans were performed with the diameter of the gear tangent to the turntable and the bore axis parallel to the table. Sphere registration was used to align these 4 scans as well. These scans were meant to capture the top and bottom of the gear.

**Figure 5:**
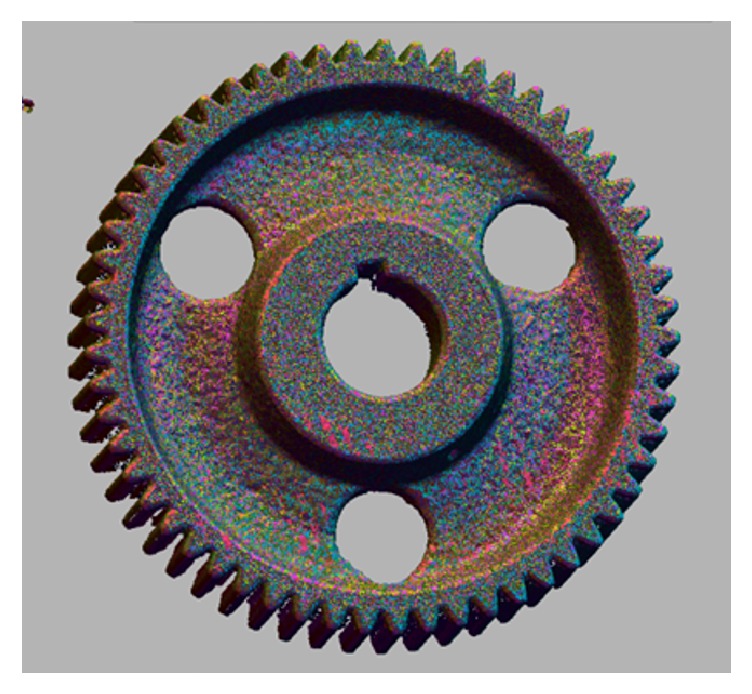
Edited point cloud before combining and sampling.

**Figure 6:**
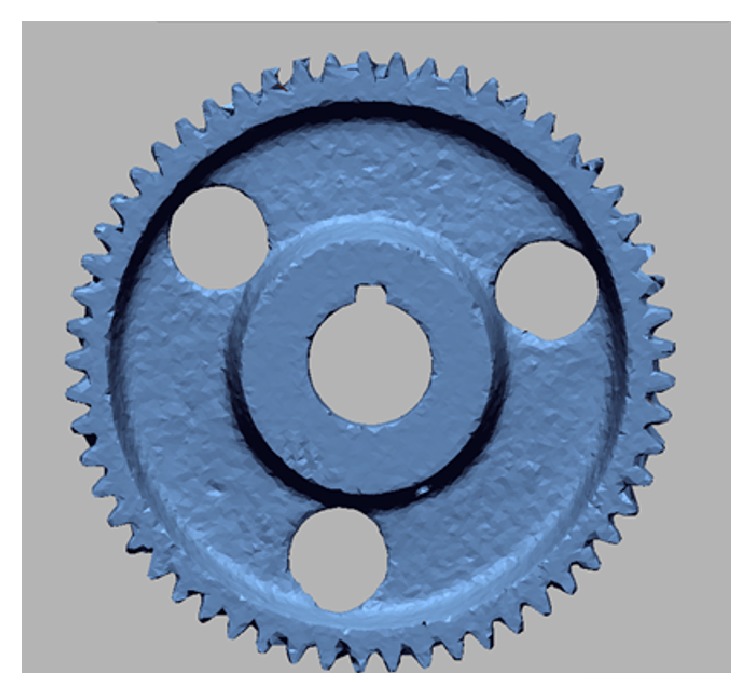
Meshed gear.

After data acquisition, the scans were imported into the scanning software for point cloud editing. The default of the capture is to import data automatically as a mesh. Therefore, it is only possible to edit the point cloud by saving the scan files first and importing the saved files as points. Once this step was completed the extraneous data was deleted. At this point, while each set of 18 scans and 4 scans were prealigned using sphere registration, it was necessary to align the horizontal and vertical scan sets to each other. This was completed in two steps. First, the scans were manually aligned based on picked points which defined position and orientation. In the second step, a global and fine alignment was used to refine the manually aligned data so that there were no steps or gaps in the point clouds. Finally, all 22 scans were combined into 1 point cloud and overlapping data was eliminated. At this point, any remaining extraneous data was filtered to eliminate noise before meshing, and the point cloud was thinned by sampling to reduce file size. After sampling and thinning the data, the distance between each point was approximately 0.003 in. Mesh generation was the final step. The software automatically created a mesh of small triangles using the points as vertices. The edited point cloud and the mesh are illustrated in Figures 5 and 6.

After completion of these steps, a solid model could be constructed in either of two ways. The model could be created through building a watertight mesh by optimizing the mesh, correcting erroneous polygons, and correcting features such as holes. From there, the model could be constructed using NURBS surfaces by setting the tolerance and the method of fit. Alternatively, the model could be parametrically modeled through sketching the outlined, meshed features and using standard CAD tools to create the gear model in what is referred to as the design intent method.

**Figure 7:**
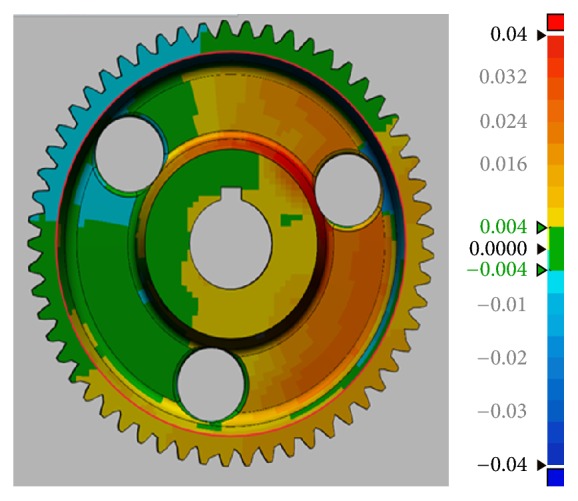
Parametric model drawn in Design X.

**Figure 8:**
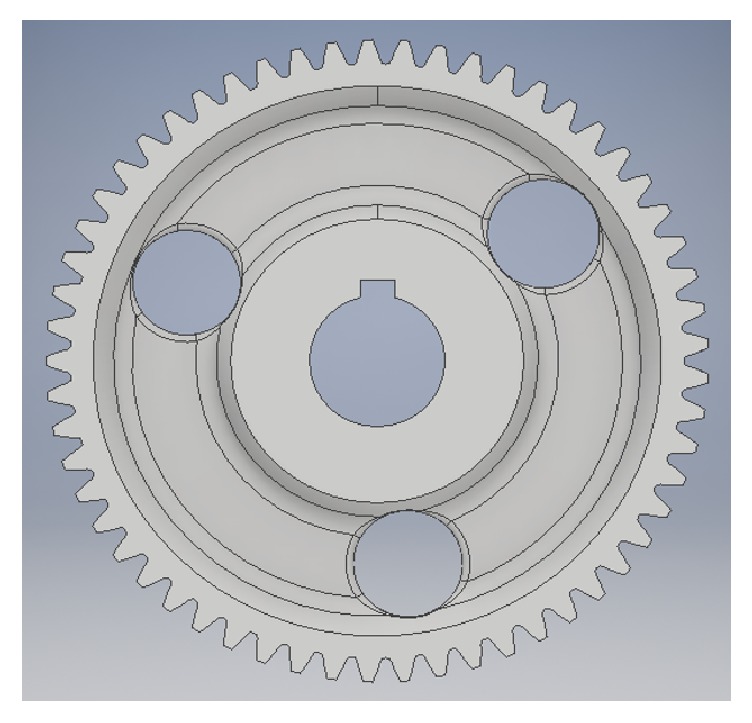
Parametric model with teeth added in Inventor.

It was the parametric modeling method that was used for constructing the gear, because of the poor quality of scan data around the teeth and keyway. The model was first aligned to the world coordinate system using reference geometry from the mesh, such as the bore and keyway of the gear, to ensure successive model parts were aligned with the scan as well as with each other. The gear was then modeled by using a combination of the scanner software and Inventor, where Inventor was used only when the average tooth depth of the gear teeth was to be modeled. The modeling process was similar to other CAD software except that the sketch of each solid was based on the outline of the polygonal mesh. The model could be transferred to Inventor for further modeling or analysis. One of the models drawn entirely in Design X is shown in Figure 7.  Figure 8 shows another model that was transferred to Inventor where the gear teeth were drawn. This process is discussed in more detail in the following sections. The completed models were exported as STEP files and subsequently converted into STL formats.

### 2.2. Printer

**Figure 9:**
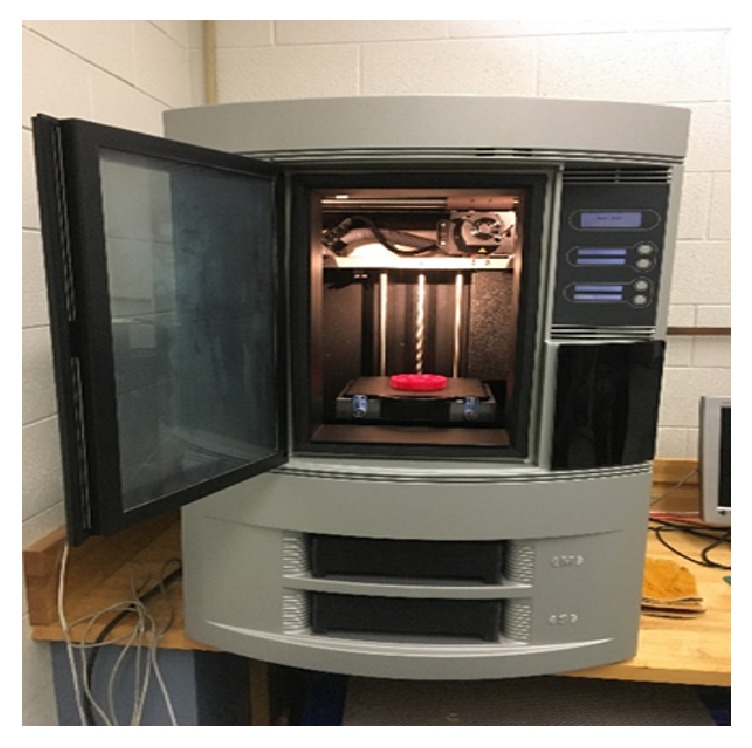
Stratasys Dimension Elite thermoplastic printer.

When printing the gear models, a Stratasys Dimension Elite thermoplastic printer (Stratasys, Ltd., MN) was used in this study. This printer, shown in Figure 9, has an enclosed heated environment with an 8 × 8 × 12 build volume, which allows it to use ABS thermoplastic. The machine has the precision to print layers between 0.007 in. and 0.01 in. thick. Each part was printed from red ABS thermoplastic at approximately a 20 to 30 percent infill density with layers of approximately 0.01 in. thickness. The plastic was extruded at a temperature between 260 and 280 degrees Celsius, while the temperature of the build chamber was maintained at approximately 75 degrees Celsius. The print speed was 0.000173 cubic in./sec., and the total time of print was 5 : 30 hours for the gear and support structure.

### 2.3. Approach 1

In Approach 1, Figure 1, in which the gear was reengineered using caliper measurements, the bore diameter and the full depth of each of the 57-tooth gear were measured using a Starret 799 caliper (L. S. Starret Co., MA) with a tolerance of 0.0005 in. The mean full tooth depth was then used to calculate the gear pitch diameter, root diameter, and outer diameter. The process of determining these dimensions started with calculating the addendum* a* and dedendum* b* of the gear teeth using (1)-(2) [19]. The diametral pitch *P* was unknown at this point; however, the full depth *h*_*t*_, available from the caliper measurements, was related to the addendum *a* and dedendum *b*, as stated in (3). Equations (1)–(3) were then combined to formulate (4). From (4), the addendum was then calculated using the full depth *h*_*t*_.

(1) a=1P

(2) b=1.25P

(3) ht=a+b

(4) ht=2.25a.

The diametral pitch was then calculated from (1) knowing the addendum. The pitch diameter* d* was found using (5) from the diametral pitch and the number of gear teeth *N*. Finally, the outer diameter *d*_*a*_ and the root diameter *d*_*b*_ were determined from the pitch diameter and the addendum and dedendum using (6) and (7). The CAD model was then drawn from these dimensions.

(5) P=Nd

(6) da=d+2a

(7) db=d−2b.

### 2.4. Approach 2

In Approach 2, Figure 1, in which the gear was reengineered using 3D scanning, the gear dimensions and shape were approximately measured by the scanner. This process is outlined in more detail in a previous section, Section 2.1. The first step in this process was to scan the gear using the capture. The next step was to edit the point cloud in Design X by merging the scans, deleting overlap, filtering noise, and finally meshing the point cloud. Once this stage was completed, the part could be modeled in Design X and simultaneously overlaid against the point cloud.

**Figure 10:**
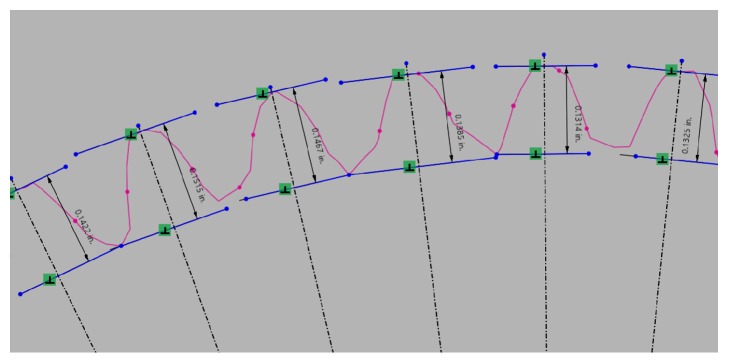
Dimensions of gear teeth as measured in the scanning software.

When modeling the gear teeth, two methods were used. In the first method, each gear tooth was modeled individually based on outlines of the mesh. The second method required measuring the full depth of each tooth in the software and taking the mean of this data. This required two assumptions. First, the gear teeth were assumed to be perfectly eccentric about the bore axis. Second, the teeth were assumed to be evenly distributed along the pitch diameter, Figure 10. Thus, a centerline could be constructed to intersect each gear tooth on the model. Each centerline was intersected by two perpendicular lines which defined what would be the top land and bottom land of each tooth. The distance between each set of lines was taken as the full depth *h*_*t*_ of each tooth. In this way, the teeth were measured, and the average was calculated. This mean was then used to calculate the required pitch diameter, root diameter, and outer diameter required to model the teeth using the relationships in (1)–(7) [19]. The model in Design X was then exported to Inventor where the teeth were constructed.

## 3. Results and Discussion

The measurements of the full depth, *h*_*t*_, of 57 teeth taken in each step in Approaches 1 and 2 were reported in terms of the mean and standard deviations in Table 1. In Approach 1, measurements were recorded for the original metal gear as well as for the printed gear. In Approach 2, the mean and standard deviations were recorded for the printed gears as well as for the meshed point cloud. The mean and standard deviation of the meshed point cloud (*μ*_*s*_, *σ*_*s*_) are taken from the model shown in Figure 7. The first gear under Approach 2 was printed based upon the model shown in Figure 7. The second gear under Approach 2 was printed based on the gear model taking *μ*_*s*_ as the tooth full depth for all 57 teeth. The associated solid model is shown in Figure 8. The mean and standard deviation of the first printed gear under Approach 2 are denoted as (*μ*_2_, *σ*_2_) while those of the second printed gear are (*μ*_2*A*_, *σ*_2*A*_). The second printed gear is shown on Figure 2(a).

Note that the terms with subscript *A* in Table 1 indicated that the gear was printed with the input solid model which was built using the averaged tooth depth. This averaging not only simplifies the process of building the solid model but also reduces the dimensional variations in the printed gear. The latter is elaborated as follows.

Let *a*_*i*_ be the dimensional parameter of the gear produced by process *i*. The difference in dimensions between two different processes, *i* and *j*, is represented by Δ_*ij*_ = *a*_*i*_ − *a*_*j*_. The mean and the standard deviation of this error are given by *μ*_Δ_*ij*__ and *σ*_Δ_*ij*__, which can be obtained by [19–21]

(8) μΔij=μai−μajσΔij=σai2+σaj21/2.

In Approach 1, the original metal gear tooth full depths are measured manually by the caliper, which are then used directly to produce the solid model. The error introduced by this process is denoted by (*μ*_*c*_, *σ*_*c*_). The mean values *μ*_*c*_ of the full depths *h*_*t*_ will be used to generate the solid model from which the gear is printed. The dimension of the printed gear is then measured with the same caliper to obtain (*μ*_1_, *σ*_1_). The mean and standard deviation of the error introduced by the 3D printing process alone can be found by using

(9) μΔc1=μc−μ1σΔc1=σc2+σ121/2.

In Approach 2, the gear is measured using a scanner. A solid model is then constructed from this scan. Finally the model is used to create a 3D print which is measured using the caliper. The mean and standard deviation of the error in the tooth full depth generated in the process from the scanned data can be found using

(10) μΔs2=μs−μ2σΔs2=σs2+σ221/2,

where the subscript* s* denotes error in the scanned data and the subscript 2 denotes error in the physical printed gear. Manually taking the average of the measured full depth of the teeth as the input to construct the solid model for printing is equivalent to setting *σ*_*c*_ or *σ*_*s*_ equal to zero. Consequently, the dimensional variations generated through the 3D printing process are reduced.

To show the significance of these errors and standard deviations, the reliabilities were calculated for a range of target values. Two scenarios are considered here. It is not known whether the true gear dimension is closer to the caliper measurement or the scan measurement. Therefore, one assumes that the true gear dimension falls into one standard deviation of the mean measured from the caliper or from the mean measured from the scanned data. Note that it amounts to 68.7% of probability in a normal distribution. The probabilities that the dimensions of the reverse engineered gears fall into this bound are tabulated in Table 2.

The results shown in Table 2 demonstrated that using the mean of the caliper measurements was more reliable than using the unaveraged scan data to reproduce a machine part such as the gear. However, using the mean of the scan measurements in Approach 2 achieved a slightly higher reliability than Approach 1 with the caliper measurements.

Scanning and reproducing each gear tooth individually, without using the mean of the measurements, produced a larger standard deviation and lower reliability. The scanner line of sight was the most significant contribution to this dimensional error in scanning. First, the scanner encountered difficulty capturing the bottom land and, in some cases, the faces of each tooth. Therefore there were fewer data points in these regions of the scan, and the resulting mesh missed key tooth dimensions. The reason for the difficulty in scanning these regions is that the scanner is based on optics. The scanner only captures what is in a direct line of sight and only if the light is reflected back toward the scanner. The areas between each gear tooth presented difficulties in this area, because they were difficult to see. This problem was significantly reduced by increasing the number of scans and the resolution of the scanner. However, the errors and reliability of the gear using the scanned data never achieved the results of the gear produced from the caliper measurements in Approach 1 unless the mean of the scan data was used.

Scanning the original gear and manually taking the average full depth in the scanner software significantly improved the error and reliability of the printed gear in Approach 2. The reliability of this approach exceeded the results in Approach 1, especially if the mean calculated from the scan data was used as the target value. It is recommended that when the scanner is used the mean of the scan data is used to construct a machine component to improve the accuracy of the reengineered product.

## 4. Conclusion

In review, this study focused on the dimensional error in reverse engineering a 57-tooth spur gear based on caliper measurements and scan measurements. The gear teeth created small and repeated features to be modeled and represented a challenge in accurately reverse engineering a complex machine component without its initial design drawing. The results showed that reverse engineering based on scanning and printing without using the mean of the data produced more errors than the process based on caliper measuring. However, calculating the mean measurement in the scan improved the reliability of the reverse engineering process.

Future work in this area includes studying the accuracy of the process in capturing complex shapes. Scanning is able to capture shapes of features that would be difficult or impossible to measure using a caliper. While this research focused on the repeated small dimensions of the gear teeth, the involute profile and the pressure angle of each tooth were not measured, and the accuracy of the shapes was not considered, which requires further study.

Finally, much research into the scanning and data processing stages must still be conducted to improve the ease and repeatability of the process. This study showed the challenges in producing many identical, recurring features from the scanning process that include human efforts and errors. The challenges stemmed from capturing, meshing, and modeling the teeth. Each of these steps relied, to some degree, on operator skill or judgement, which allowed some dimensional variability. For example, the data collection for each tooth was slightly different for each of the 57 teeth. Therefore, after processing and modeling each gear tooth separately, there was variation in the model. While this study suggested taking an average of the already processed data to remedy this problem, improving data acquisition and processing to solve the problem is left for further research.

## Figures and Tables

**Table 1:** The mean and standard deviation of tooth full depths.

Gear	Tooth full-depth mean (*μ*)	Tooth full-depth standard deviation (*σ*)
Original metal gear (*μ*_*c*_, *σ*_*c*_)	0.1422 in.	0.0018 in.
*Approach 1*		
Reverse engineered gear (Avg.) (*μ*_1*A*_, *σ*_1*A*_)	0.1439 in.	0.0017 in.
*Approach 2*		
Meshed point cloud in Design X (*μ*_*s*_, *σ*_*s*_)	0.1406 in.	0.0053 in.

Reverse engineered gear (no Avg.) (*μ*_2_, *σ*_2_)	0.1399 in.	0.0062 in.
Reverse engineered gear (Avg.) (*μ*_2*A*_, *σ*_2*A*_)	0.1425 in.	0.0026 in.

**Table 2:** Reliabilities in Approaches 1 and 2.

Approach	% reliability based on caliper	% reliability based on Scanner
Approach 1 (Avg.)	49.54	87.91
Approach 2 (no Avg.)	16.91	48.56
Approach 2 (Avg.)	50.82	90.29
